# Supplementary material for: Lateral septum DREADD activation alters male prairie vole prosocial and antisocial behaviors, not partner preferences
Source: Commun Biol. 2022 Nov 26;5:1299. doi: 10.1038/s42003-022-04274-z (PMC9701193; doi:10.1038/s42003-022-04274-z)
Supplement: Supplementary file 1 — Supplemental Material [file 42003_2022_4274_MOESM1_ESM.pdf]

Supplementary Information for

Lateral septum DREADD activation alters male prairie vole prosocial and antisocial behaviors, not partner preferences

Lindsay L. Sailer, Ashley H. Park, Abigail Galvez, Alexander G. Ophir*

*Corresponding author. Email: ophir@cornell.edu

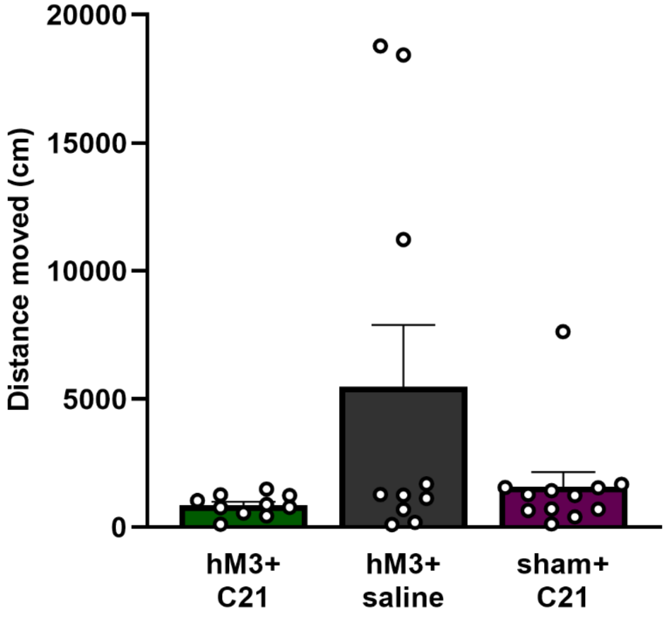

Supplementary Figure 1 | LS activation does not affect distance moved. Distance moved (centimeters; cm) by the hM3+C21 (green bars), hM3+saline (grey bars), and sham+C21 (purple bars) subjects during the social approach test. Data are presented as mean ± SEM. Dots represent individual data for hM3+C21 (n = 10), hM3+saline (n = 10), and sham+C21 (n = 12) subjects. (LMM, treatment effect: $F_{(2,29)} = 3.19$, $p = 0.06$).

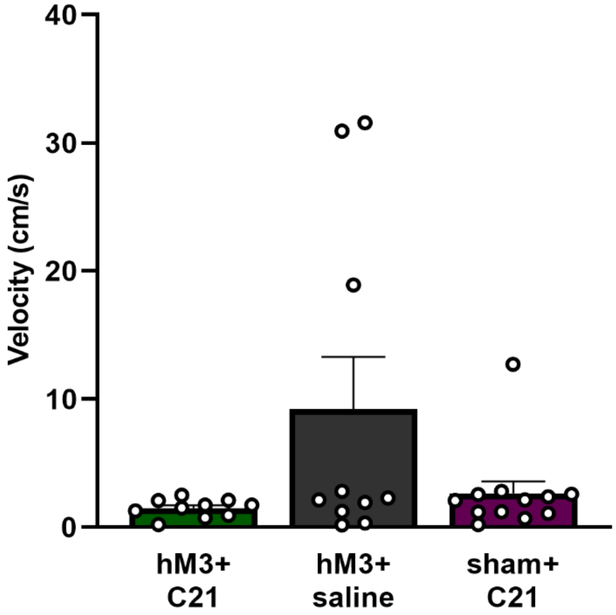

Supplementary Figure 2 | LS activation does not affect velocity. Velocity (centimeters/second; cm/s) of the hM3+C21 (green bars), hM3+saline (grey bars), and sham+C21 (purple bars) subjects during the social approach test. Data are presented as mean ± SEM. Dots represent individual data for hM3+C21 (*n* = 10), hM3+saline (*n* = 10), and sham+C21 (*n* = 12) subjects. (LMM, treatment effect: $F_{(2,29)} = 3.21$, $p = 0.06$).

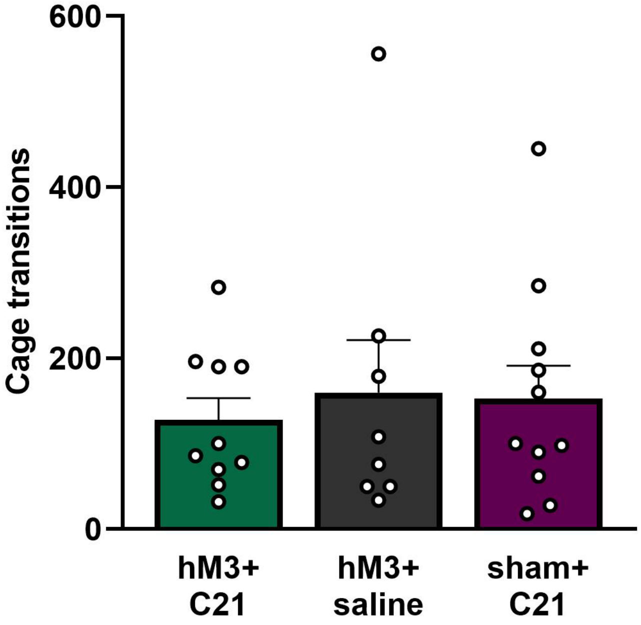

Supplementary Figure 3 | LS activation does not affect cage transitions. Cage transitions of the hM3+C21 (green bars), hM3+saline (grey bars), and sham+C21 (purple bars) subjects during the partner preference test. Data are presented as mean ± SEM. Dots represent individual data for hM3+C21 (*n* = 10), hM3+saline (*n* = 8), and sham+C21 (*n* = 11) subjects.
