# Supplementary material for: Lateral septum DREADD activation alters male prairie vole prosocial and antisocial behaviors, not partner preferences
Source: Commun Biol. 2022 Nov 26;5:1299. doi: 10.1038/s42003-022-04274-z (PMC9701193; doi:10.1038/s42003-022-04274-z)
Supplement: Supplementary file 2 — Description of Additional Supplementary Files [file 42003_2022_4274_MOESM2_ESM.pdf]

Description of Additional Supplementary Files

File name: Supplementary Data 1

Description: Source Data for Figure 2a

File name: Supplementary Data 2

Description: Source Data for Figure 2b

File name: Supplementary Data 3

Description: Source Data for Figure 2

File name: Supplementary Data 4

Description: Source Data for Figure 3

File name: Supplementary Data 5

Description: Source Data for Figure 3b

File name: Supplementary Data 6

Description: Source Data for Figure 3c

File name: Supplementary Data 7

Description: Source Data for Figure 4a

File name: Supplementary Data 8

Description: Source Data for Figure 4b

File name: Supplementary Data 9

Description: Source Data for Figure 4c

File name: Supplementary Data 10

Description: Source Data for Figure 4d

File name: Supplementary Data 11

Description: Source Data for Figure 4e

File name: Supplementary Data 12

Description: Source Data for Figure 4f

File name: Supplementary Data 13

Description: Source Data for Supplementary Figure 1

File name: Supplementary Data 14

Description: Source Data for Supplementary Figure 2

File name: Supplementary Data 15

Description: Source Data for Supplementary Figure 3
