# Supplementary material for: Lateral septum DREADD activation alters male prairie vole prosocial and antisocial behaviors, not partner preferences
Source: Commun Biol. 2022 Nov 26;5:1299. doi: 10.1038/s42003-022-04274-z (PMC9701193; doi:10.1038/s42003-022-04274-z)
Supplement: Supplementary file 18 — Reporting Summary [file 42003_2022_4274_MOESM18_ESM.pdf]

Corresponding author(s): Alexander G. Ophir
Last updated by author(s): Nov 8, 2022

Reporting Summary

Nature Portfolio wishes to improve the reproducibility of the work that we publish. This form provides structure for consistency and transparency in reporting. For further information on Nature Portfolio policies, see our Editorial Policies and the Editorial Policy Checklist.

Statistics

For all statistical analyses, confirm that the following items are present in the figure legend, table legend, main text, or Methods section.

n/a	Confirmed	
☐	☒	The exact sample size (*n*) for each experimental group/condition, given as a discrete number and unit of measurement
☐	☒	A statement on whether measurements were taken from distinct samples or whether the same sample was measured repeatedly
☐	☒	The statistical test(s) used AND whether they are one- or two-sided<br>*Only common tests should be described solely by name; describe more complex techniques in the Methods section.*
☐	☒	A description of all covariates tested
☐	☒	A description of any assumptions or corrections, such as tests of normality and adjustment for multiple comparisons
☐	☒	A full description of the statistical parameters including central tendency (e.g. means) or other basic estimates (e.g. regression coefficient) AND variation (e.g. standard deviation) or associated estimates of uncertainty (e.g. confidence intervals)
☐	☒	For null hypothesis testing, the test statistic (e.g. *F*, *t*, *r*) with confidence intervals, effect sizes, degrees of freedom and *P* value noted<br>*Give P values as exact values whenever suitable.*
☒	☐	For Bayesian analysis, information on the choice of priors and Markov chain Monte Carlo settings
☒	☐	For hierarchical and complex designs, identification of the appropriate level for tests and full reporting of outcomes
☐	☒	Estimates of effect sizes (e.g. Cohen's *d*, Pearson's *r*), indicating how they were calculated

Our web collection on statistics for biologists contains articles on many of the points above.

Software and code

Policy information about availability of computer code

Data collection	All behavioral procedures were manually scored by an observer blind to treatment using Noldus Ethovision XT 13 (Noldus, Leesburg, VA, USA), Noldus Observer XT 11, or BORIS 7.9.7.
Data analysis	Data were analyzed with RStudio (version 1.2.1335) using a linear mixed-model (LMM) framework with the packages lme4, emmeans, and lmerTest.

For manuscripts utilizing custom algorithms or software that are central to the research but not yet described in published literature, software must be made available to editors and reviewers. We strongly encourage code deposition in a community repository (e.g. GitHub). See the Nature Portfolio guidelines for submitting code & software for further information.

Data

Policy information about availability of data

All manuscripts must include a data availability statement. This statement should provide the following information, where applicable:

- Accession codes, unique identifiers, or web links for publicly available datasets
- A description of any restrictions on data availability
- For clinical datasets or third party data, please ensure that the statement adheres to our policy

Numerical source data for graphs presented in the main figures and supplementary figures are included in the Supplementary Data file.

Human research participants

Policy information about studies involving human research participants and Sex and Gender in Research.

Reporting on sex and gender	N/A
Population characteristics	N/A
Recruitment	N/A
Ethics oversight	N/A

Note that full information on the approval of the study protocol must also be provided in the manuscript.

Field-specific reporting

Please select the one below that is the best fit for your research. If you are not sure, read the appropriate sections before making your selection.

☒ Life sciences ☐ Behavioural & social sciences ☐ Ecological, evolutionary & environmental sciences

For a reference copy of the document with all sections, see nature.com/documents/nr-reporting-summary-flat.pdf

Life sciences study design

All studies must disclose on these points even when the disclosure is negative.

Sample size	Samples sizes were determined based on previous publications.
Data exclusions	All animals were included in the analyses (n = 10-12 animals/treatment group), with the exception of the data presented for the partner preference test. Two subjects from the hM3+Saline group and one sham+C21 subject group were not included because of technical difficulties with recording equipment.
Replication	Reproducibility of the experimental findings were verified by performing surgeries and testing cohorts of subjects.
Randomization	Subjects from different breeding pairs were randomly assigned to treatment groups.
Blinding	Investigators were blind to group allocation during data collection (scoring of behavioral videos).

Reporting for specific materials, systems and methods

We require information from authors about some types of materials, experimental systems and methods used in many studies. Here, indicate whether each material, system or method listed is relevant to your study. If you are not sure if a list item applies to your research, read the appropriate section before selecting a response.

Materials & experimental systems

n/a	Involved in the study	
☒	☐	Antibodies
☒	☐	Eukaryotic cell lines
☒	☐	Palaeontology and archaeology
☐	☒	Animals and other organisms
☒	☐	Clinical data
☒	☐	Dual use research of concern

Methods

n/a	Involved in the study	
☒	☐	ChIP-seq
☒	☐	Flow cytometry
☒	☐	MRI-based neuroimaging

Animals and other research organisms

Policy information about studies involving animals; ARRIVE guidelines recommended for reporting animal research, and Sex and Gender in Research

Laboratory animals	Prairie voles (90-120 days old) from breeding pairs that were offspring of wild caught animals captured in Champagne County, Illinois, USA.

March 2021

Wild animals	N/A
Reporting on sex	Findings apply to only one sex . All subjects were unrelated, and sexually mature males. Several studies have shown there are large sex differences in the lateral septum of prairie voles. Males have a much denser vasopressin fiber network in the lateral septum. Moreover, we have shown that parental variation causes male prairie voles to exhibit impaired social behavior and increased vasopressin receptor gene expression in the lateral septum. All animals were included in the analyses (n = 10-12 animals/treatment group).
Field-collected samples	N/A
Ethics oversight	Institutional Animal Care and Use Committee (IACUC) of Cornell University (protocol number 2013-0102).

Note that full information on the approval of the study protocol must also be provided in the manuscript.
